# Receiver Diversity Combining Using Evolutionary Algorithms in Rayleigh Fading Channel

**DOI:** 10.1155/2014/128195

**Published:** 2014-06-19

**Authors:** Mohsen Akbari, Mohsen Riahi Manesh, Ayman A. El-Saleh, Ahmed Wasif Reza

**Affiliations:** ^1^Department of Electrical Engineering, Faculty of Engineering, University of Malaya, 50603 Kuala Lumpur, Malaysia; ^2^Faculty of Engineering, Multimedia University, 63100 Cyberjaya, Selangor, Malaysia

## Abstract

In diversity combining at the receiver, the output signal-to-noise ratio (SNR) is often maximized by using the maximal ratio combining (MRC) provided that the channel is perfectly estimated at the receiver. However, channel estimation is rarely perfect in practice, which results in deteriorating the system performance. In this paper, an imperialistic competitive algorithm (ICA) is proposed and compared with two other evolutionary based algorithms, namely, particle swarm optimization (PSO) and genetic algorithm (GA), for diversity combining of signals travelling across the imperfect channels. The proposed algorithm adjusts the combiner weights of the received signal components in such a way that maximizes the SNR and minimizes the bit error rate (BER). The results indicate that the proposed method eliminates the need of channel estimation and can outperform the conventional diversity combining methods.

## 1. Introduction

Diversity techniques are among the prominent ways to improve the reliability of wireless communication systems [1, 2]. These techniques, which fundamentally amount to transmitting signals over independent fading channels, are used in reality to fight against fading. The main idea of diversity is to extract information from the received signal components transmitted over multiple fading channels to improve the received signal-to-noise ratio (SNR) [3, 4]. The large-enough spacing is essential in order to make sure that the received signals are independent, which is a vital requisite to acquire the full benefit of the diversity receiver [5]. It is obvious that there would be a small probability that all the received versions of signal are in a deep fade. Therefore, these techniques assume independent fading effects over the different signal paths. Out of the three mechanisms, namely, path loss, large scale, and small scale fading, the first two are somehow similar and can be mitigated by the power control over a long period of time. Diversity techniques are particularly intended to overcome the small scale fading.

**Figure 1:**
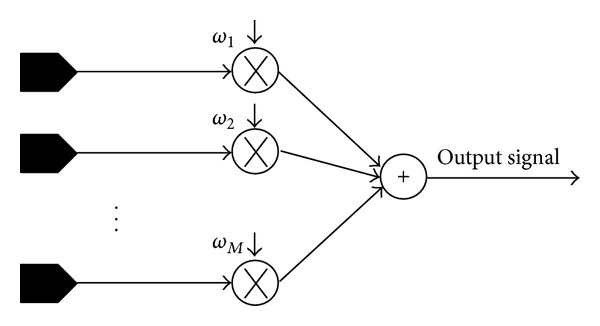
Diversity combining block diagram.

In the past decades, different kinds of diversity receivers functioning over a variety of fading channels have been comprehensively reviewed in the literature [5]. The widely used diversity techniques include maximal ratio combining (MRC), equal gain combining (EGC), and selection combining (SC) [6, 7]. The aim of these techniques is to find a set of weights $\vec{\omega }=\left[\omega_{1},\omega_{2},\ldots ,\omega_{M}\right]$, as shown in Figure 1, which optimizes a specific objective function. Here, the weights are selected to minimize the effect of fading on the received multiple signal components for each individual user. In MRC, the received signals are weighted accordingly so that the SNR at the output of the combiner is the sum of the average SNR of each branch. In EGC, on the other hand, the received signals are weighted equally and then added. In SC, the branch with the highest SNR is selected. In all cases, we consider that the receiver has the necessary information of channel fading.

The performance of these methods has been extensively examined in the literature for Rayleigh fading. If the channel is perfectly estimated at the receiver, MRC can be applied to maximize the output SNR and minimize the bit error rate (BER) [8]. However, since the channel estimation is often imperfect in practice, the estimation error will decay the system performance. While this problem has long been investigated [9, 10], the recent evolutions in mobile communication systems have renewed the attention in comprehending and mitigating the effect of imperfect channel estimation on diversity techniques [11]. The error performance of MRC in Rayleigh fading environment with independent and identically distributed (i.i.d.) diversity branches is investigated in [12]. In [13], the SNR distribution is given for similar scenarios. In [14], the error performance of MRC with independent but not identically distributed (i.n.d.) branches is studied. In [15, 16], a comparison of hybrid SC/MRC scheme with SC and MRC schemes over Rayleigh fading channels in two scenarios of flat and exponentially decaying multipath intensity profile (MIP) has been done. In [17], the hybrid diversity scheme is studied as such selection combining and MRC are at the first and second stages, respectively. In [18], *L* out of *N* diversity branches was selected and combined using MRC over Rayleigh fading channel. The performance study of conventional MRC receiver in the presence of cochannel interference has also been a substantial interest of researchers [19–24]. Particularly in [24], the effect of the number of interferers on the diversity gain has been investigated in the context of frequency-selective Rayleigh fading. The study, however, has been done with the assumption of the prefect channel estimation of a desired user, which may not be the case in practice. The impact of imperfect channel estimation on the performance of diversity receivers in noise-limited circumstances has been presented in [25–30]. However, considering the frequency-nonselective fading, the investigation has been widened to circumstances with multiple cochannel interferers [31–33].

In this paper, to overcome the effect of imperfect estimation of channel state information, a diversity combining technique based on the imperialistic competitive algorithm (ICA) is proposed in which the signals received by the antennas are iteratively weighted based on ICA operation. The channel model used is slow flat Rayleigh fading. It should be noted that Rayleigh model is the simplest and the most controllable model, but it is not effective in all circumstances. However, since this paper basically aims at studying the use of evolutionary algorithms on receiver diversity, the authors believe that Rayleigh model is enough. Hence, the results given in this paper are only a ballpark figure of pros and cons of diversity methods and different algorithms to improve them. It is shown that the proposed combining method does not require the channel estimation, and it outperforms the MRC when channel estimation is imperfect. On the other hand, it has almost the same performance as MRC when channel estimation is assumed to be perfect. The ICA method shows faster convergence speed when compared with particle swarm optimization- (PSO-) and genetic algorithm- (GA-) based methods. This makes ICA a promising solution for the real-time applications.

## 2. System Model

In this paper, it is assumed that the information bits are modulated by binary phase-shift keying (BPSK) modulation. The channel is assumed to be frequency nonselective and slowly fading over the length of the transmitted symbol. We also assume that *M* diversity branches are employed at the receiver for reception. In addition, this research work assumes that the diversity branches are sufficiently far apart from each other, so that the received signals are statistically independent with negligible correlation. This is a vital requisite to acquire the full advantage of the diversity receiver [5]. The received signal at the *i*th branch is given by

(1) $$\begin{array}{c} r_{i}\left(t\right)=g_{i}S\left(t\right)+n_{i},\;i=1,2,\ldots ,M, \end{array}$$

where *S*(*t*) is the unit-power transmitted signal and *g*_*i*_ denotes the complex channel gain with uncorrelated and Gaussian distributed real and imaginary parts, each with zero mean and variance *σ*_*g*_*i*__^2^. The noise random variable *n*_*i*_ is complex additive white Gaussian noise (AWGN) with zero mean and variance *σ*_*n*_^2^ = *N*_0_/2. The channel gain *g*_*i*_ at two different diversity branches is assumed to be identically distributed. It is also assumed that *g*_*i*_ and *n*_*i*_ are uncorrelated. The signal power over one symbol period *T*_*s*_, at *i*th path, is

(2) $$\begin{array}{c} p=\frac{1}{T_{s}}\overset{T_{s}}{\underset{0}{\int }}{\left|g_{i}\right|}^{2}{\left|S(t)\right|}^{2}dt={\left|g_{i}\right|}^{2}\frac{1}{T_{s}}\overset{T_{s}}{\underset{0}{\int }}{\left|S(t)\right|}^{2}dt={\left|g_{i}\right|}^{2}. \end{array}$$

Since we are assuming slow fading, the term |*g*_*i*_|^2^ remains constant over a symbol period and can be taken out of the integral. *S*(*t*) is assumed to have unit power. As a result, the instantaneous SNR at the *i*th path is

(3) $$\begin{array}{c} \gamma_{i}=\frac{{\left|g_{i}\right|}^{2}}{\sigma_{n}^{2}}. \end{array}$$

Since we are considering Rayleigh fading, *g*_*i*_ = |*g*_*i*_|*e* ^*j∠g*_*i*_^ where *∠g*_*i*_ is uniformly distributed over [2*π*, 0] and *g*_*i*_ has a Rayleigh pdf. Therefore, |*g*_*i*_|^2^ and hence *γ*_*i*_ have exponential pdf. Consider

(4) $$\begin{array}{c} \left|g_{i}\right|\sim \frac{2\left|g_{i}\right|}{P_{0}}e^{{-|g_{i}|}^{2}/P_{0}},\\ {\;\gamma }_{i}\sim \frac{1}{\Gamma }e^{-\gamma_{i}/\Gamma },\\ \Gamma =E\left\{\gamma_{i}\right\}=\frac{E\left\{{\left|g_{i}\right|}^{2}\right\}}{\sigma_{n}^{2}}=\frac{P_{0}}{\sigma_{n}^{2}}. \end{array}$$

*P*_0_ is the statistical average of |*g*_*i*_|^2^ and Γ represents the average SNR at each individual branch, which serves as a basic parameter to improve the SNR at the receiver.

The bit error rate (BER) in a BPSK system, given an SNR of *γ*_*i*_, is identified by $\mathrm{erfc}\sqrt{2\gamma_{i}}$, where $\mathrm{erfc}\left(x\right)=(2/\sqrt{\pi })\int_{x}^{\infty }e^{-t^{2}}dt$ [12]. Therefore, the BER averaged over the Rayleigh fading in (4) is given by [13]

(5) BER=∫0∞2|gi|P0e−|gi|2/P0erfc⁡(2|gi|σn)d(|gi|)=12(1−Γ1+Γ).

The physical model assumes the fading to be independent from one branch to the next. Each branch, therefore, acts as an independent sample of the random fading process (here, Rayleigh). It means each branch receives an independent copy of the transmitted signal. Our goal here is to combine these independent samples in a way to achieve the desired goal of increasing the SNR and reducing the BER.

## 3. Conventional Weighting Schemes

In this section, different combining schemes, such as selection combining (SC), equal gain combining (EGC) and maximal ratio combining (MRC) are investigated.

### 3.1. Selection Combining

In selection combining (SC), the branch with the greatest SNR is chosen as output SNR to be used in the next step:

(6) $$\begin{array}{c} \omega_{i}=\{\begin{array}{cc} 1 & \gamma_{i}=\mathrm{Max}\\ 0 & \text{otherwise}. \end{array} \end{array}$$

The average output SNR for SC is defined as [14]

(7) $$\begin{array}{c} \gamma_{T}=\Gamma \overset{M}{\underset{i=1}{\sum }}\frac{1}{i}≅\Gamma \left(C-\ln M+\frac{1}{2M}\right), \end{array}$$

in which *C* is Euler's constant. The final approximation is valid for *M* ≥ 3. The overall BER is obtained by bringing together the conditional BER at a certain SNR. In BPSK modulation, the conditional BER is $\mathrm{erfc}\sqrt{2\gamma_{T}}$ and the total BER is

(8) $$\begin{array}{c} {\text{BER}}_{T}=\overset{\infty }{\underset{0}{\int }}erfc\left(\sqrt{2\gamma_{T}}\right)\frac{M}{\Gamma }e^{\gamma_{T}/\Gamma }{\left[1-e^{\gamma_{T}/\Gamma }\right]}^{M-1}d\gamma_{T}. \end{array}$$

### 3.2. Equal Gain Combining

Equal gain combiner (EGC) sets unit gain at each branch to increase the average SNR in the system. In the equal gain combiner,

(9) ωi=ej∠gi  ⟹ωi∗gi=|gi|  ⟹  ω→G→T=∑i=0M−1|gi|, G→=[g1,g2,…,gM],γi=[∑i=0M−1|gi|]2Mσn2,γT=E{[∑i=0M−1|gi|]}2Mσn2=[1+(M−1)π4]Γ.

There is no closed form solution for the BER for general *M*, but several researchers have investigated the BER performance in several kinds of fading channels [15, 16].

### 3.3. Maximal Ratio Combining

In MRC, receiver linearly combines the received signal *r*_*i*_(*t*) with *ω*_*i*_, which is the weighting coefficient of the *i*th branch. The output signal *r*(*t*) of the linear diversity combiner is then given by

(10) $$\begin{array}{c} r\left(t\right)=\overset{M}{\underset{i=1}{\sum }}\omega_{i}r_{i}\left(t\right)=S\left(t\right)\overset{M}{\underset{i=1}{\sum }}\omega_{i}g_{i}+\overset{M}{\underset{i=1}{\sum }}\omega_{i}n_{i}. \end{array}$$

Since *S*(*t*) is assumed to have unit power, SNR at the output of combiners is

(11) $$\begin{array}{c} \gamma_{T}\left(\vec{\omega }\right)=\frac{1}{\sigma_{n}^{2}}\frac{{\left|\sum_{i=1}^{M}\omega_{i}g_{i}\right|}^{2}}{\sum_{i=1}^{M}{\left|\omega_{i}\right|}^{2}}=\frac{{\left|\vec{\omega }{\vec{G}}^{T}\right|}^{2}}{E\left\{{\left|\vec{\omega }{\vec{N}}^{T}\right|}^{2}\right\}}, \end{array}$$

(12) $$\begin{array}{c} E\left\{{\left|\vec{\omega }{\vec{N}}^{T}\right|}^{2}\right\}=E\left\{\left|\vec{\omega }{\vec{N}}^{T}{\vec{\omega }}^{T}\vec{N}\right|\right\}=\sigma_{n}^{2}{\vec{\omega }}^{2}. \end{array}$$

According to the Cauchy-Schwarz inequality, MRC with perfect channel estimation has maximum output SNR among all methods if $\vec{\omega }$ is linearly proportional to $\vec{G}$. If $\vec{\omega }=\vec{G}\Rightarrow \gamma_{T}={\left|\vec{G}{\vec{G}}^{T}\right|}^{2}/\sigma_{n}^{2}{\vec{G}}^{T}\vec{G}=\vec{G}{\vec{G}}^{T}/\sigma_{n}^{2}\Rightarrow \gamma_{T}=\sum_{i=1}^{M}\left|\gamma_{i}\right|$, the output SNR is, therefore, the sum of the SNR at each element. By using the above assumption, the expected value of the output SNR is therefore *M* times the SNR at each branch.

For the case of imperfect channel estimation, which is the main issue in practice, it is observable that the SNR is highly dependent on *ω*_*i*_. Therefore, the optimal solution is the weighting vector, which maximizes the objective function *γ*_*T*_ in (11). We assume *p*_*i*_ is the estimate of the complex gain *g*_*i*_ on the *i*th diversity branch and *e*_*i*_ is the estimation error with zero mean and variance *σ*_*e*_^2^ = *σ*_*g*_^2^(1 − *ρ* ^2^) where *ρ* ∈ [0, 1] is the normalized estimation error correlation coefficient. Under Gaussian error model, *g*_*i*_ and *p*_*i*_ are related as *g*_*i*_ = *p*_*i*_ + *e*_*i*_ [16]. According to the diversity combining rule, the combiner's weights take on the *ω*_*i*_ = *p*_i_* for MRC diversity, which is based on the Cauchy-Schwartz inequality, maximizes (11) if the channel is perfectly estimated (i.e., *ρ* = 1). However, since channel estimation is often imperfect in practice, the MRC is a suboptimal solution [17–37].

## 4. Evolutionary Algorithm-Based Weighting Schemes

In this paper, the optimization problem is to maximize the output SNR of the combiner $\gamma_{T}(\vec{\omega })$ in (11) where $\vec{\omega }=[\omega_{1},\omega_{2},\ldots ,\omega_{M}]$ and *M* is the number of variables (number of branches) of $\gamma_{T}(\vec{\omega })$ with *ω* ^*l*^ ≤ *ω* ≤ *ω* ^*u*^ where *ω* ^*l*^ = 0 and *ω* ^*u*^ = 1 are lower and upper limits on *ω*, respectively. Thus, we propose to use evolutionary algorithms at the combiner so that all possible weighting vectors $\vec{\omega }$ are investigated and the optimal one, which maximizes the output SNR in (11), is obtained. Hence, the need for estimating the channel state information is eliminated. As mentioned earlier, a simple Rayleigh channel model is satisfactory to illustrate the efficiency of the method. We mainly introduce an imperialist competitive algorithm (ICA) to find the optimal $\vec{\omega }$ and compare its performance with two other iterative algorithms, namely, genetic algorithm (GA) and particle swarm optimization (PSO) to prove its effectiveness. The three algorithms of GA, PSO, and ICA are presented in the next sections.

### 4.1. Genetic Algorithm-Based Weighting Scheme

In the genetic algorithm (GA), a group of chromosomes will be arbitrarily generated. Equation (11) is used as the fitness function to evaluate the SNR of randomly generated chromosomes of the initial population. Then, a new population from the former population will be reproduced based on the fitness scores (output SNR values) of its chromosomes and the process is repeated until a predefined termination criterion is met [36]. Better populations can be continually formed due to the concept of surviving the fit/best chromosomes. In GA terminology, the evolutionary process of forming an offspring population from a parent population is called generation [37]. The number of produced generations is predetermined by the designer or self-set based on the quality of obtainable solutions. The algorithm is configured to maximize the SNR and it is outlined as follows.

**Step 1 .**

Randomly generate a population of* pops* chromosomes.

**Step 2 .**

Decode each chromosome into its corresponding weighting vector ${\vec{\omega }}_{j}={\left[\omega_{j1},\omega_{j2},\ldots ,\omega_{jM}\right]}^{T}$, where *ω*_*ji*_ ∈ [0,1], *i* = 1, 2,…, *M*, and *j* = 1, 2,…, *pops*.

**Step 3 .**

Compute the SNR value of every decoded weighting vector ${\vec{\omega }}_{j}$ using (11) and rank and identify the best ⌊*pops*∗*elite*⌋ chromosomes that have maximized SNR.* elite* is a parameter that determines a fraction of* pops*, that is, *elite* ∈ [0,1), and ⌊·⌋ denotes the floor operation.

**Step 4 .**

After large-enough generations (runs of the algorithm), if the output SNR of the system converges to a stable value at each iteration, the procedure is terminated. Otherwise, increase the generation number by one.

**Step 5 .**

Reproduce ⌈*pops*∗(1 − *elite*)⌉ new chromosomes where ⌈·⌉ denotes ceiling operation, and construct new population by concatenating the newly ⌈*pops*∗(1 − *elite*)⌉ reproduced chromosomes with the best ⌊*pops*∗*elite*⌋ found in Step 3. Jump to Step 2.

Finally, the optimal weighting vector (decoded chromosomes) that leads to the highest stable value of the output SNR can be indicated and used.

### 4.2. Particle Swarm Optimization-Based Weighting Scheme

PSO algorithm is abstracted from the social behavior of swarm of fish and birds. The behavior of these social organizations is emulated by the PSO algorithm. Each particle in PSO algorithm functions based on its own knowledge as well as the group knowledge and has two main features: position and velocity. In each iteration, information about the best position is cooperatively exchanged among the particles. The steps involved in the PSO algorithm are as follows.

**Step 1 .**

Randomly generate *N* number of particle positions (weighting vectors) as ${\vec{\omega }}_{s}={\left[\omega_{1},\omega_{2},\ldots ,\omega_{M}\right]}^{T}$, (*s* = 1,…, *N*) and *N* number of length-*M* velocity vectors ${\vec{v}}_{s}^{\left(j\right)}$, which are initially set to zero. Here, particle position and velocity at iteration *j* are demonstrated by ${\vec{\omega }}_{s}^{\left(j\right)}$ and ${\vec{v}}_{s}^{\left(j\right)}$, respectively.

**Step 2 .**

Calculate the objective function (SNR in (11)) for particle positions as $\gamma_{T}\left({\vec{\omega }}_{1}^{\left(j\right)}\right)$, $\gamma_{T}\left({\vec{\omega }}_{2}^{\left(j\right)}\right)\cdots \gamma_{T}\left({\vec{\omega }}_{N}^{\left(j\right)}\right)$. Find the maximum SNR and name its corresponding position as *P*_best,*j*_. The best experienced particle position among all iterations is called global best position and is expressed by *Gl*_best_.

**Step 3 .**

Update the velocity of the particles by

(13) $$\begin{array}{c} {\vec{v}}_{s}^{\left(j\right)}={\vec{v}}_{s}^{\left(j-1\right)}+c_{1}r_{1}\left[P_{\text{best},j}-{\vec{\omega }}_{s}^{\left(j-1\right)}\right]+c_{2}r_{2}\left[{Gl}_{\text{best}}-{\vec{\omega }}_{s}^{\left(j-1\right)}\right], \end{array}$$

where individual and social learning acceleration coefficients are, respectively, denoted by *c*_1_ and *c*_2_ and *r*_1_ and *r*_2_ which are the random numbers between 0 and 1.

**Step 4 .**

Update the position of particles as follows:

(14) $$\begin{array}{c} {\vec{\omega }}_{s}^{\left(j\right)}={\vec{\omega }}_{s}^{\left(j-1\right)}+{\vec{v}}_{s}^{\left(j\right)}. \end{array}$$

**Step 5 .**

Check the convergence. The output SNR in (11) is regularly checked at each iteration. After a large-enough number of iterations, if the algorithm results in the same output SNR in each iteration, the procedure is terminated. Otherwise, set *j* = *j* + 1 and the process is repeated from Step 2.

Therefore, the value of the *Gl*_best_ is the optimal weighting vector that maximizes the SNR at the output of the combiner.

### 4.3. Modified Imperialist Competitive Algorithm-Based Weighting Scheme

It is considerably obvious that genetic and physical evolution does not happen as fast as the communal and the academic evolution of human being. Due to this fact, some developing algorithms have applied the cultural side of social life in order to reach well outcomes. Imperialistic competition and human's sociopolitical evolution inspire ICA [38–40]. ICA algorithm has not been deep rooted in refining diversity combining issue to the best knowledge of the author. Hence, checking the effectiveness of the algorithm in comparison to other techniques is the main disquiet of this research. The main steps of ICA are explained as follows.

**Step 1 .**

Generate *N*_pop_ numbers of countries (combiner's weighting vector shown in Figure 1) as ${\vec{\omega }}_{k}={\left[\omega_{1},\omega_{2},\ldots ,\omega_{M}\right]}^{T}$ where *k* = 1,…, *N*_pop_. The SNR value of each country, based on (11), is calculated and sorted.

**Step 2 .**

**Figure 2:**
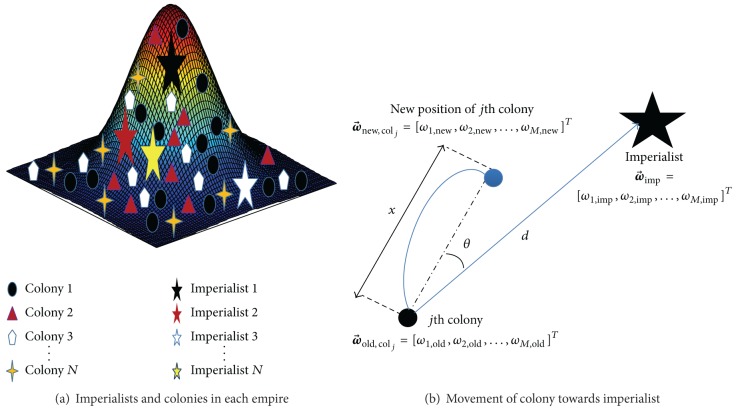
Current and future position of imperialists and colonies in imperialistic competitive algorithm.

*N*_imp_ of most powerful (in terms of SNR) countries are chosen as imperialists to form empires and the rest of *N*_col_ countries are called colonies.  Figure 2(a) depicts the initial colonies for each empire. The initial number of colonies for an empire is randomly selected from *N*_col_ with respect to the empire's imperialist power (*p*_imp_), which is its corresponding normalized SNR:

(15) Initial  number  of  colonies  in  an  empire =Nc=round(Ncol·pimp).

**Step 3 .**

Colonies in an empire start to move in the search space towards an imperialist state in different directions (assimilation). ${\vec{x}}_{j}={\left[x_{1},x_{2},\ldots ,x_{M}\right]}^{T}\;\;\left(j=1,\ldots ,N_{c}\right)$ is the transferred distance of the *j*th colony, which is randomly chosen from the interval of [0→,Υ′·d→j] where $\vec{0}$ is a 1-by-*M* zero vector, ′*Υ* is the assimilation coefficient (0 < ′*Υ* ≤ 2), and ${\vec{d}}_{j}{=\left[d_{1},d_{2},\ldots ,d_{M}\right]}^{T}$ is the distance between the imperialist and *j*th colony in an empire, which is calculated by

(16) d→j=ω→imp−ω→colj=[ω1,imp−ω1,colj,ω2,imp −ω2,colj,…,ωM,imp−ωM,colj]T.

Therefore, the new position of the *j*th colony is calculated as follows [41]:

(17) $$\begin{array}{c} {\vec{\omega }}_{{\text{new},\text{col}}_{j}}={\vec{\omega }}_{{\text{old},\text{col}}_{j}}+{\vec{x}}_{j}+\vec{r}\cdot \tan\left(\theta \right), \end{array}$$

where $\vec{r}$ is a 1-by-*M* random vector, whose values are uniformly distributed on (−1, +1) and *θ* is assimilation deviation which can be chosen from −*π*/2 < *θ* < *π*/2.  Figure 2(b) depicts how colonies transfer to their related imperialist.

**Step 4 .**

The cost of each colony in the new position is again computed based on (11). Position exchange between a colony and imperialist can happen in this step. In other words, if a colony in its new position has a higher SNR than that of the imperialists, it has the chance to take the control of empire by replacing the existing imperialist. Consider

(18) γT(ω→new,colj)>γT(ω→imp) ⟹jth  colony  will  become  the  impersialist.

**Step 5 .**

Imperialistic competition is being performed in this step. The colony with the lowest SNR value from the empire with the weakest power is chosen and provided to one of the best empires. The total power (in terms of SNR) of an empire is calculated as follows:

(19) $$\begin{array}{c} \text{Total empire's power}=\gamma_{T}\left({\vec{\omega }}_{\text{imp}}\right)+\xi \left(\frac{\sum_{j=1}^{N_{c}}\gamma_{T}\left({\vec{\omega }}_{{\text{col}}_{j}}\right)}{N_{c}}\right), \end{array}$$

where positive number, *ξ*, is equal to or less than one (0 < *ξ* ≤ 1).

**Step 6 .**

When all colonies of an empire move to other powerful empires and just imperialist remains, this imperialist automatically joins best empire as a simple colony. This empire will then be removed.

**Step 7 .**

Stop condition will satisfy, if only one empire remains. In other words, after a while, only one empire with the highest total power (as in (19)) remains, which controls all the colonies. In this condition, all of the colonies and the imperialists have the same position (weighting vector) and cost (SNR at (11)). Otherwise, algorithm jumps to Step 3.

The equivalent weighting vector of the final imperialist is the best vector that maximizes the output SNR of our diversity problem here.

**Figure 3:**
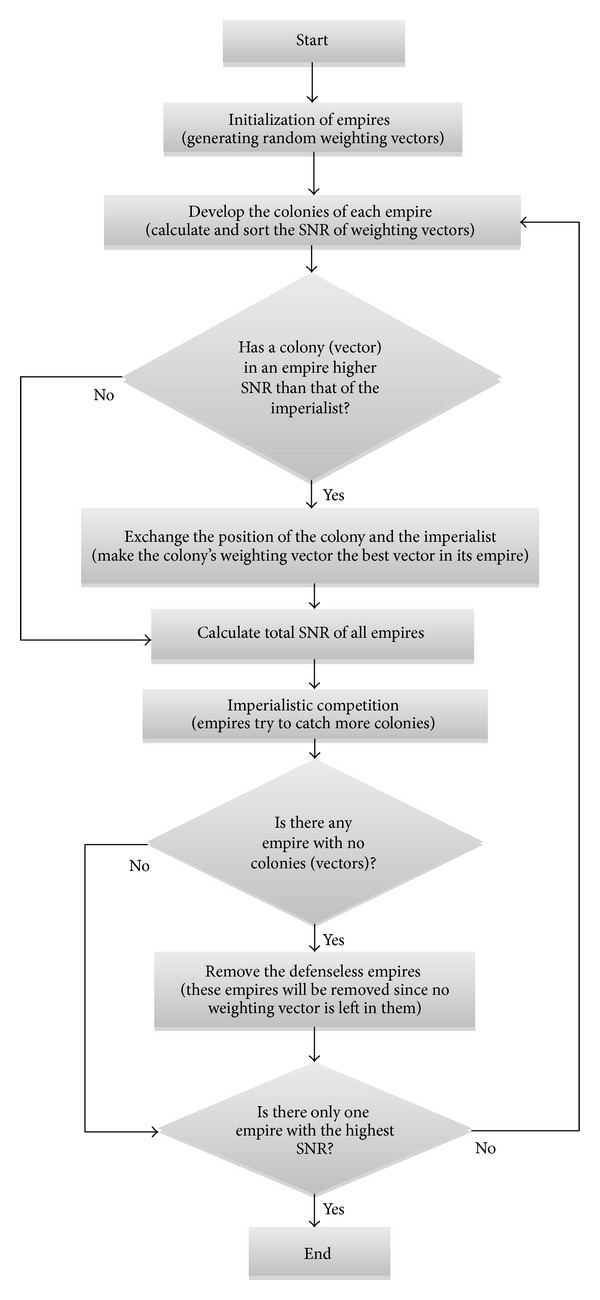
ICA-based flowchart that maximizes the quality of the received signal in diversity combining technique.

Figure 3 abstractly shows the flowchart of ICA, which explains how ICA is applied to improve the reliability of the wireless communication systems.

## 5. Numerical Results and Discussion

**Figure 4:**
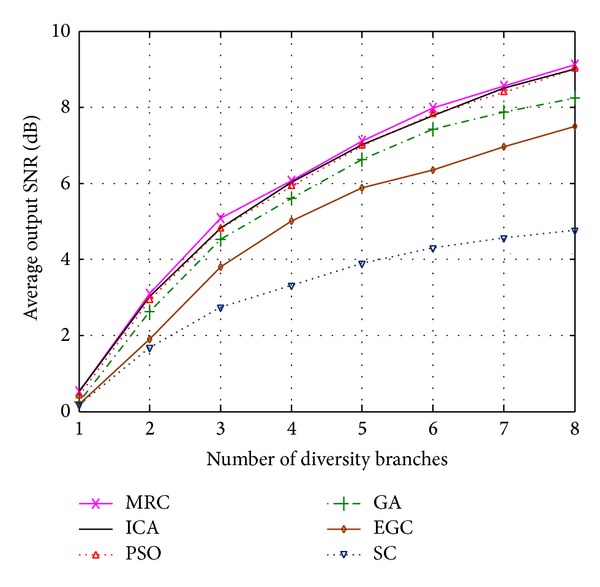
Normalized output SNR of MRC-, ICA-, PSO-, GA-, EGC-, and SC-based methods when the channel is perfectly estimated.

In this section, Monte-Carlo simulation is employed to present the performance of the proposed ICA-based diversity combining technique and compare it with PSO, GA, MRC, EGC, and SC methods in two different scenarios of the perfect and imperfect channel estimation. It is assumed that the average symbol energy *E*_*s*_ = 1 and channel gain and AWGN variances are *σ*_*g*_^2^ = *σ*_*n*_^2^ = 0.5 per dimension. The parameters for the PSO are *N* = 25 and *c*_1_ = *c*_2_ = 2.  Figure 4 compares the normalized output SNR of ICA-, PSO-, and GA-based combining with MRC, EGC, and SC in terms of different numbers of diversity branches when the channel is perfectly estimated (*ρ* = 1) [42]. As expected, it has been observed that the MRC provides the best performance when channel estimation is perfect. However, the ICA- and PSO-based solutions demonstrate almost the same SNR gain as MRC without the need for channel estimation. Since the parameters in each algorithm are generally problem-dependent, the* set-and-test approach* is used in this work to obtain the optimal values for them. In other words, *c*_1_ *r*_1_ and *c*_2_ *r*_2_ in PSO or *θ*, ′*Υ* in ICA guarantee that the particles or colonies would fly over the target about half the time. In this respect, the environment has been tested separately for parameters as mentioned in Table 1 and the optimal value is found in Table 2.

**Figure 5:**
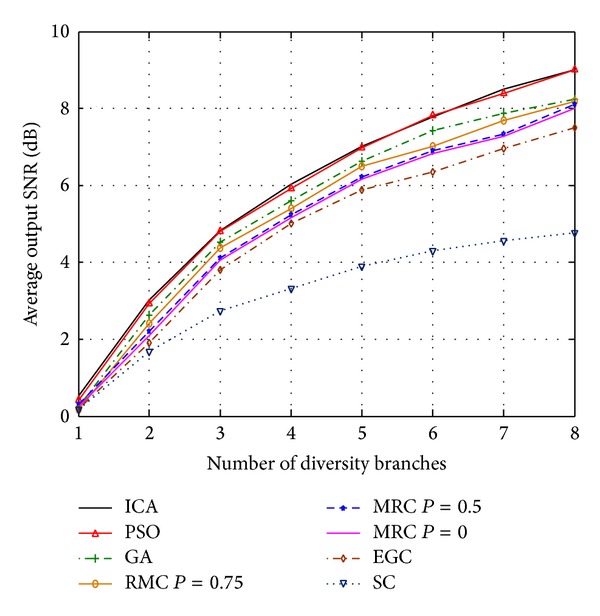
Comparison of normalized output SNR of ICA-, PSO-, GA-, MRC-, EGC-, and SC-based methods for imperfect channel estimation.

**Figure 6:**
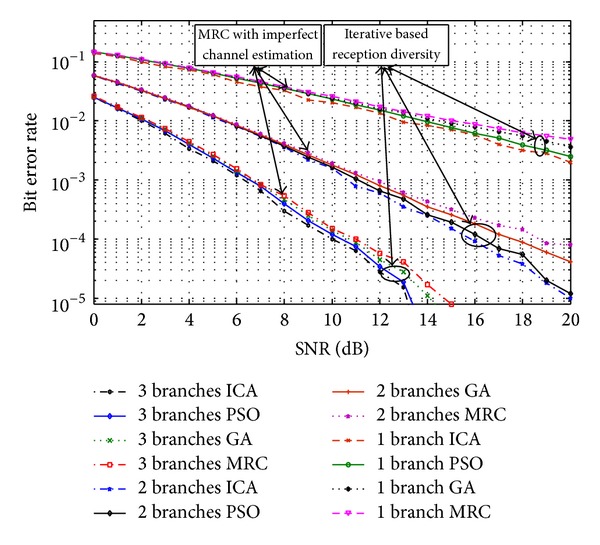
Error performance of ICA-, PSO-, and MRC-based methods for different numbers of diversity branches.

**Figure 7:**
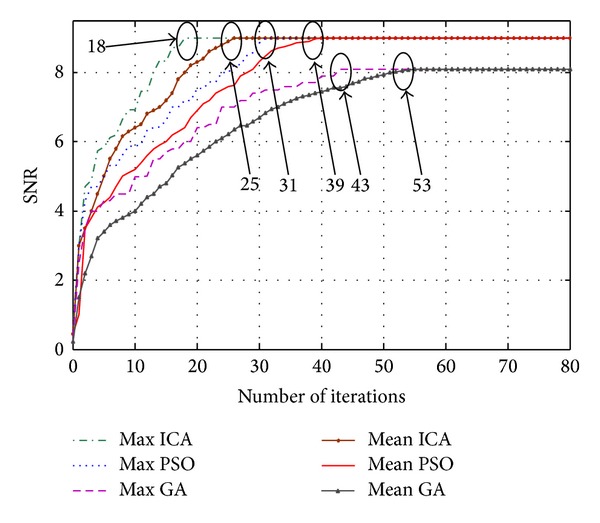
Convergence performance of the iterative algorithms.

The comparison between the iterative based algorithms and MRC methods in the case of imperfect channel estimation (*ρ* = 0, 0.5, 0.75) [43, 44] is illustrated in Figure 5. It can be seen that ICA- and PSO-based methods outperform MRC when channel estimation is imperfect. The achieved improvement can be justified by the ability of the algorithms to investigate the search space thoroughly and evaluate the objective function in (11) to maximize the output SNR. As it is shown in Figures 4 and 5, PSO and ICA results are quite close to each other. However, on the other hand, Figures 6 and 7 present the superiority of ICA over PSO in terms of achievable BER and SNR, respectively. These two metrics declare that the quality of the diversity performance achieved by ICA is quite better than that of PSO. However, *t*-test has been carried out to provide an evidence of statistical significance in the difference of means of these two algorithms. With a significance level of 0.10, it has been found that the two-tailed *P* value is 0.0805, which means that the results are considered statistically significant.

Considering the BPSK modulation and imperfect channel estimation, the error performance of the MRC-, ICA- and PSO-based methods for 1, 2, and 3 diversity branches is illustrated in Figure 6. It is observable that the bit error rate of the ICA-based technique is considerably lower than that of the MRC. For instance, for a two-branch diversity, the MRC approximately requires almost 3 dB higher SNR than that of ICA-based to achieve a *BER* = 10^−4^. In addition, as it is shown, increasing the number of branches results in improved error performance.

Next, Figure 7 compares the convergence of ICA, PSO, and GA algorithms used in the diversity method. The number of diversity branches is assumed to be 8. The mean and max of each algorithm are achieved when the algorithms run for 100 times. The average of all results is called mean and the best one among these 100 simulations, which results in the maximum output SNR, is named as max. As it is shown in the figure, max curve in ICA method converges after 18 iterations whereas about 31 iterations of PSO algorithm are needed for convergence. This indicates the higher convergence speed of the ICA compared to PSO.

Table 3 shows the details of convergence speed for each method. The term NA in Table 3 indicates that the iteration number for that specific condition is not available. For instance, ICA-based method with 5 countries cannot converge in 100 iterations. Moreover, the number of fitness evaluations as a parameter to compare the complexity of iterative algorithms has been provided in Table 3. The number of fitness evaluations is simply the product of the number of generations by which the maximum SNR fitness is achieved multiplied by the number of fitness evaluations performed in every iteration. The latter equals the population size of any of these algorithms. For instance, with ICA, the number of iterations required to achieve the maximum SNR is 18 and the number of countries is 25. This means that the number of fitness evaluations to find the optimal setting is 450, which is considerably low with the advancement of signal processing and computing cores.

The SNR variances of ICA, PSO, and GA are shown in Table 4 and are recorded every five iterations until the 55th iteration after which the variances are zeroed when all colonies, particles, and chromosomes of ICA, PSO, and GA, respectively, converge to the same optima. Considering the values in the table and calculating standard deviations at each iteration, one can conclude that ICA, with all of its fluctuations around its mean, can still outperform the other two algorithms. This validates the superiority of this algorithm in comparison with the other methods.

## 6. Conclusion

One of the most important issues in reception antenna diversity occurs when the channel is imperfectly estimated. This defective estimation results in obtaining a vector of the weighting coefficient of the combiner that deteriorates the SNR and BER performance of the system at the receiver. To address the issue, an ICA-based diversity combining method is proposed to optimize the weighting vector, which is used to combine the received signals at the receiver. Simulation results validate that the proposed method provides better SNR and error performance than that of other evolutionary algorithms, such as GA and PSO and conventional MRC when channel estimation is imperfect. On the other hand, in the perfect channel estimation environment, the proposed method performs as effectively as the MRC.

## Figures and Tables

**Table 1:** Different parameters values used for testing.

GA		PSO		ICA	
Population size	10, 20, 30, 40, 50	Population size	5, 10, 15, 20, 25, 50	Population size	5, 10, 15, 20, 25, 35

Mutation rate	0.01, 0.1, 0.15, 0.2, 0.3, 0.4, 0.5, 0.6	Learning coefficients	1.8, 1.85, 1.9, 1.95, 2, 2.05, 2.1	Mean colonies power coefficient	0 < *ξ* ≤ 1

Crossover rate	0.5, 0.65, 0.75, 0.85, 0.95	*r*_1_ and *r*_2_	*U*(0, 1)	Assimilation coefficient	0 < Υ ≤ 2

Population for reproduction rate	0.5, 0.6, 0.7, 0.8, 0.9				

**Table 2:** Optimal parameter values for ICA, PSO, and GA algorithms which maximize the output SNR.

GA		PSO		ICA	
Population size	50	Population size	25	Population size	25
Mutation rate	0.3	Learning coefficients	2	Mean colonies power coefficient	0.15
Crossover rate	0.95	*r*_1_ and *r*_2_	*U*(0, 1)	Assimilation coefficient	1.7
Population for reproduction rate	0.9				

**Table 3:** Performance comparison of ICA and PSO assisted for different population numbers.

ICA						PSO					
Number of countries	5	10	15	20	25	Number of particles	5	10	15	20	25
Average output SNR (*N* = 8)	8.53	8.78	9.09	9.15	9.21	Average output SNR (*N* = 8)	8.46	8.63	8.96	9.06	9.13
Max convergence iterations	89	72	53	29	18	Max convergence iterations	92	74	52	38	31
Mean convergence iterations	NA	92	68	43	25	Mean convergence iterations	NA	96	88	47	39
Number of fitness evaluations	445	720	795	580	450	Number of fitness evaluations	460	740	780	760	775

**Table 4:** Variance of SNR of all algorithms when the population size is 25.

Iteration number	5	10	15	20	25	30	35	40	45	50	55
ICA	0.75	0.21	0.98	0.57	0.0004	0	0	0	0	0	0
PSO	0.65	0.64	0.38	0.24	0.28	0.62	0.04	0	0	0	0
GA	0.98	1	0.79	0.65	0.46	0.28	0.17	0.14	0.05	0.01	0

## References

[1] Simon MK, Alouini MS (2005). *Digital Communication over Fading Channels*.

[2] Skraparlis D, Sakarellos VK, Panagopoulos AD, Kanellopoulos JD (2009). Performance of N-branch receive diversity combining in correlated lognormal channels. *IEEE Communications Letters*.

[3] Karagiannidis GK, Zogas DA, Sagias NC, Kotsopoulos SA, Tombras GS (2005). Equal-gain and maximal-ratio combining over nonidentical weibull fading channels. *IEEE Transactions on Wireless Communications*.

[4] Annavajjala R, Milstein LB (2005). Performance analysis of linear diversity-combining schemes on Rayleigh fading channels with binary signaling and Gaussian weighting errors. *IEEE Transactions on Wireless Communications*.

[5] Simon MK, Alouini M-S (2005). *Digital Communication over Fading Channels*.

[6] Sakarellos VK, Skraparlis D, Panagopoulos AD, Kanellopoulos JD (2011). Cooperative diversity performance of selection relaying over correlated shadowing. *Physical Communication*.

[7] Kong N Performance comparison among conventional selection combining, optimum selection combining and maximal ratio combining.

[8] Lee EA, Messerschmitt DG (2004). *Digital Communications*.

[9] Mallik RK, Winters JH (2010). Deterministic linear combining receivers for random fading channels. *IEEE Transactions on Communications*.

[10] Al-Qahtani FS, Zummo SA, Gurung AK, Hussain ZM (2010). Spectral efficiency of maximum ratio combining (MRC) over slow fading with estimation errors. *Digital Signal Processing*.

[11] Ahn KS, Heath RW (2009). Performance analysis of maximum ratio combining with imperfect channel estimation in the presence of cochannel interferences. *IEEE Transactions on Wireless Communications*.

[12] Roy S, Fortier P (2004). Maximal-ratio combining architectures and performance with channel estimation based on a training sequence. *IEEE Transactions on Wireless Communications*.

[13] Verdu S (1998). *Multiuser Detection*.

[14] Godara LC (2001). *Handbook of Antennas in Wireless Communications*.

[15] Thompson JS Antenna array performance with channel estimation errors.

[16] You R, Li H, Bar-Ness Y (2005). Diversity combining with imperfect channel estimation. *IEEE Transactions on Communications*.

[17] Kong N, Eng T, Milstein LB A selection combining schemefor RAKE receivers.

[18] Eng T, Kong N, Milstein LB (1996). Comparison of diversity combining techniques for Rayleigh-fading channels. *IEEE Transactions on Communications*.

[19] Win MZ, Winters JH (1999). Analysis of hybrid selection/maximal-ratio combining in Rayleigh fading. *IEEE Transactions on Communications*.

[20] Dinamani A, Das S, Bijendra L, Shruti R, Babina S, Kiran B Performance of a hybrid MRC/SC diversity receiver over Rayleigh fading channel.

[21] Cui J, Sheikh AUH (1999). Outage probability of cellular radio systems using maximal ratio combining in the presence of multiple interferers. *IEEE Transactions on Communications*.

[22] Aalo VA, Zhang J (1999). On the effect of cochannel interference on average error rates in Nakagami-fading channels. *IEEE Communications Letters*.

[23] Shah A, Haimovich AM (2000). Performance analysis of maximal ratio combining and comparison with optimum combining for mobile radio communications with cochannel interference. *IEEE Transactions on Vehicular Technology*.

[24] Peña-Martin JP, Romero-Jerez JM, Aguilera G, Goldsmith AJ (2009). Performance comparison of MRC and IC under transmit diversity. *IEEE Transactions on Wireless Communications*.

[25] Zhang X, Beaulieu NC (2007). Explicit analytical expressions for outage and error rate of diversity cellular systems in the presence of multiple interferers and correlated Rayleigh fading. *IEEE Transactions on Communications*.

[26] Radaydeh RM (2009). MRC in the presence of asynchronous cochannel interference over frequency-selective Rayleigh fading channels. *IEEE Transactions on Vehicular Technology*.

[27] Gans MJ (1971). The effect of Gaussian error in maximal ratio combiners. *IEEE Transactions on Communications*.

[28] Annavajjala R, Milstein LB (2005). Performance analysis of linear diversity-combining schemes on Rayleigh fading channels with binary signaling and Gaussian weighting errors. *IEEE Transactions on Wireless Communications*.

[29] Ma Y, Schober R, Zhang D (2007). Exact BER for M-QAM with MRC and imperfect channel estimation in Rician fading channels. *IEEE Transactions on Wireless Communications*.

[30] Najafizadeh L, Tellambura C (2006). BER analysis of arbitrary QAM for MRC diversity with imperfect channel estimation in generalized Ricean fading channels. *IEEE Transactions on Vehicular Technology*.

[31] Annavajjala R, Cosman PC, Milstein LB (2007). Performance analysis of linear modulation schemes with generalized diversity combining on Rayleigh fading channels with noisy channel estimates. *IEEE Transactions on Information Theory*.

[32] Gifford WM, Win MZ, Chiani M (2008). Antenna subset diversity with non-ideal channel estimation. *IEEE Transactions on Wireless Communications*.

[33] Roy S, Fortier P (2004). Maximal-ratio combining architectures and performance with channel estimation based on a training sequence. *IEEE Transactions on Wireless Communications*.

[34] Tokgoz Y, Rao BD (2006). The effect of imperfect channel estimation on the performance of maximum ratio combining in the presence of cochannel interference. *IEEE Transactions on Vehicular Technology*.

[35] Ahn KS, Heath RW (2009). Performance analysis of maximum ratio combining with imperfect channel estimation in the presence of cochannel interferences. *IEEE Transactions on Wireless Communications*.

[36] Annavajjala R, Milstein LB (2005). Performance analysis of linear diversity-combining schemes on Rayleigh fading channels with binary signaling and Gaussian weighting errors. *IEEE Transactions on Wireless Communications*.

[37] Akbari M, Hossain MR, Manesh MR, El-Saleh AA, Kareem AM (2012). Minimizing sensing decision error in cognitive radio networks using evolutionary algorithms. *KSII Transactions on Internet and Information Systems*.

[38] Khabbazi A, Atashpaz-Gargari E, Lucas C (2009). Imperialist competitive algorithm for minimum bit error rate beamforming. *International Journal of Bio-Inspired Computation*.

[39] Nazari-Shirkouhi S, Eivazy H, Ghodsi R, Rezaie K, Atashpaz-Gargari E (2010). Solving the integrated product mix-outsourcing problem using the imperialist competitive algorithm. *Expert Systems with Applications*.

[40] Niknama T, Taherian Fard E, Pourjafarian N, Rousta A (2011). An efficient hybrid algorithm based on modified imperialist competitive algorithm and K-means for data clustering. *Engineering Applications of Artificial Intelligence*.

[41] Kaveh A, Talatahari S (2010). Optimum design of skeletal structures using imperialist competitive algorithm. *Computers and Structures*.

[42] Ma Y, Jin J (2007). Effect of channel estimation errors on M-QAM With MRC and EGC in Nakagami fading channels. *IEEE Transactions on Vehicular Technology*.

[43] Ismail MH, Matalgah MM (2006). Performance of dual maximal ratio combining diversity in nonidentical correlated Weibull fading channels using Padé approximation. *IEEE Transactions on Communications*.

[44] di Renzo M, Guidotti A, Corazza GE (2013). Average rate of downlink heterogeneous cellular networks over generalized fading channels: a stochastic geometry approach. *IEEE Transactions on Communications*.

